# Identification and Validation of Selected Universal Stress Protein Domain Containing Drought-Responsive Genes in Pigeonpea (*Cajanus cajan* L.)

**DOI:** 10.3389/fpls.2015.01065

**Published:** 2016-01-06

**Authors:** Pallavi Sinha, Lekha T. Pazhamala, Vikas K. Singh, Rachit K. Saxena, L. Krishnamurthy, Sarwar Azam, Aamir W. Khan, Rajeev K. Varshney

**Affiliations:** ^1^Center of Excellence in Genomics (CEG), International Crops Research Institute for the Semi-Arid Tropics (ICRISAT)Hyderabad, India; ^2^School of Plant Biology and the Institute of Agriculture, The University of Western AustraliaPerth, WA, Australia

**Keywords:** *in-silico* analysis, drought responsive genes, expression profiling, pigeonpea, legumes

## Abstract

Pigeonpea is a resilient crop, which is relatively more drought tolerant than many other legume crops. To understand the molecular mechanisms of this unique feature of pigeonpea, 51 genes were selected using the Hidden Markov Models (HMM) those codes for proteins having close similarity to universal stress protein domain. Validation of these genes was conducted on three pigeonpea genotypes (ICPL 151, ICPL 8755, and ICPL 227) having different levels of drought tolerance. Gene expression analysis using qRT-PCR revealed 6, 8, and 18 genes to be ≥2-fold differentially expressed in ICPL 151, ICPL 8755, and ICPL 227, respectively. A total of 10 differentially expressed genes showed ≥2-fold up-regulation in the more drought tolerant genotype, which encoded four different classes of proteins. These include plant U-box protein (four genes), universal stress protein A-like protein (four genes), cation/H(+) antiporter protein (one gene) and an uncharacterized protein (one gene). Genes *C.cajan_29830* and *C.cajan_33874* belonging to *uspA*, were found significantly expressed in all the three genotypes with ≥2-fold expression variations. Expression profiling of these two genes on the four other legume crops revealed their specific role in pigeonpea. Therefore, these genes seem to be promising candidates for conferring drought tolerance specifically to pigeonpea.

## Introduction

Abrupt climate changes and unavailability of sufficient water supply can severely affect the productivity of agriculturally important crops. Additionally, frequent exposure of environmental stresses such as drought is adversely affecting the plant growth and yield. Drought can occur at any stage of plant growth and the degree of yield loss depends on the onset time, intensity and duration of stress (Hu and Xiong, 2014). Pigeonpea is usually grown under marginal environments that are often subjected to water stress at different stages of growth and development. Even for short-duration varieties, yield gets affected due to water stress during late flowering and early pod development stages (Lopez et al., 1996). During seed hardening, the crop requires considerable amount of water and at this crucial stage unavailability of water often causes terminal drought. Despite having a deeper root system, drought is still one of the major yield-limiting factors, especially at critical seedling and reproductive stages of pigeonpea (Saxena, 2008). There has been a rousing progress made in developing drought-tolerant pigeonpea genotypes, but still it is difficult to meet the conditions arisen due to climate change. It is feasible to develop drought tolerant varieties through genomics-assisted breeding that would facilitate yield stability under water-deficient conditions (Varshney et al., 2014).

Since drought is a complex trait and is controlled by multigenes, identification of candidate genes and understanding the molecular mechanism associated with drought tolerance in pigeonpea is critical. Many studies have been carried out in model plants to identify candidate genes associated with drought response (see Mir et al., 2012). In pigeonpea, ample amount of genomics resources has been developed which can be deployed to identify candidate drought tolerant genes specific to pigeonpea. Moreover, the pigeonpea genome sequence reported 111 homologous sequences corresponding to universal drought-responsive protein sequences from the Viridiplantae (Varshney et al., 2012). Similarly, the development of comprehensive transcriptome assembly (Kudapa et al., 2012) and the identification of genes involved in abiotic stresses tolerance have been reported (Priyanka et al., 2010; Sekhar et al., 2010; Saxena et al., 2011; Deeplanaik et al., 2013).

Functional characterization of genes involved in different stress-responsive pathways such as photosynthesis and carbohydrate metabolism (Basu et al., 1999), related to stress-responsive transcription factors (Nakashima et al., 2009), signal transduction and regulatory compounds (Ramanjulu and Bartels, 2002; Sreenivasulu et al., 2007) gives an insight into the mechanisms adopted by plants to cope with drought stress. In this context, using bioinformatics approach, a total of 32 drought-responsive ESTs were retrieved from seven plant genera namely, *Glycine, Hordeum, Manihot, Medicago, Oryza, Pinus*, and *Triticum* (Isokpehi et al., 2011). Similarly, in soybean, 32 drought responsive genes involved in 17 metabolic pathways were identified and were validated in pigeonpea to know their association with drought stress (Deeplanaik et al., 2013).

To identify differentially expressed genes, many technologies such as microarray, DNA chip-based array, genome-wide transcript profiling, and quantitative real-time PCR (qRT-PCR) have been deployed in a number of studies (Ozturk et al., 2002; Degenkolbe et al., 2009; Lenka et al., 2011). qRT-PCR is the most commonly used approach for expression analysis of genes in many crop species including pigeonpea (Borges et al., 2012; Qiao et al., 2012; Deeplanaik et al., 2013; Recchia et al., 2013; Turyagyenda et al., 2013; Da Silva et al., 2015; Sinha et al., 2015).

The present study involves *in-silico* identification of selected universal stress protein domain containing drought-responsive genes. The qRT-PCR validation of these genes was carried out on pigeonpea genotypes with different levels of drought tolerance. Drought stress was imposed on all the selected genotypes and compared with well-watered controls to validate the candidate genes involved with drought tolerance in pigeonpea. The genes were also validated using a tolerant and a susceptible genotype each from four legumes namely, chickpea, groundnut, common bean, and cowpea. The identified candidate genes in future, can be functionally validated using transgenic approaches. Additionally to utilize the identified drought tolerant genes, markers can be developed using haplotype analysis approach, which will accelerate crop yield even under drought stress conditions.

## Materials and methods

### Plant materials

Three genotypes, ICPL 227, ICPL 8755, and ICPL 151, which are the parents of two mapping populations segregating for drought tolerance, were used. ICPL 151 and ICPL 8755 are known to have a low-level of drought tolerance as compared to ICPL 227 (Lopez et al., 1996; Saxena et al., 2011). To validate the putative pigeonpea drought-responsive candidate genes in other legumes, one tolerant and one susceptible genotype of each legume crop namely, chickpea (ICC 4958, tolerant and ICC 1882, susceptible), groundnut (CSMG 84-1, tolerant and ICGS 76, susceptible), common bean (BAT 477, tolerant and DOR 364, susceptible), and cowpea (IT93K503-1, tolerant and UC-C B46, susceptible) were selected, respectively (Table 1).

**Table 1:** **Details of genotypes used for expression analysis**.

**Genotypes**	**Features**	**References**
**PIGEONPEA**		
ICPL 151	Less drought tolerant	Saxena et al., 2011
ICPL 8755	Less drought tolerant	Saxena et al., 2011
ICPL 277	More drought tolerant	Saxena et al., 2011
**CHICKPEA**		
ICC 4958	Tolerant	Varshney et al., 2014
ICP 1882	Susceptible	Varshney et al., 2014
**GROUNDNUT**		
CSMG 84-1	Tolerant	Gautami et al., 2012
ICGS-76	Susceptible	Gautami et al., 2012
**COMMON BEAN**		
BAT 477	Tolerant	Galindo et al., 2007
DOR 364	Susceptible	Galindo et al., 2007
**COWPEA**		
IT93K503-1	Tolerant	Barrera-Figueroa et al., 2011
UC-C B46	Susceptible	Barrera-Figueroa et al., 2011

### Drought stress treatment and tissue harvesting

Seeds were thoroughly washed with DEPC treated water, sown in 3 inches plastic pots (one seed per pot) filled with autoclaved black soil, sand, and vermicompost (10:10:1 v/v) mixture. All the plants were grown under controlled conditions in three replications. For imposing drought stress, slow drought (dry down) stress was imposed on the plants when they reached 22 days old seedling stage. A calculated amount of water was added to each pot, which was weighed at regular intervals. Control plants were maintained throughout at 80% relative water content (RWC) whereas stressed plants were dried down gradually to 20% RWC. The intensity of the drought stress was measured by recording the transpiration ratio (TR) on a daily basis. Stressed plants were allowed to dry through transpiration until the TR reached 0.1. Root tissues were harvested from the stressed plants from all three replicates. The root samples were gently wiped with 70% ethanol to remove soil particles. All tissues were immediately frozen in liquid nitrogen and stored at −80°C for RNA isolation.

### RNA isolation and cDNA synthesis

Total RNA was isolated from all the frozen root samples using TRIzol (Invitrogen, USA) and was purified using DNase (Qiagen, GmbH, Germany) through an RNeasy Plant Mini kit according to the manufacturer's instructions. The concentration of total RNA was checked using NanoDropND-1000 (NanoDrop Technologies, USA) and RNA integrity was assessed on 1% denaturing formaldehyde agarose gel. cDNA was prepared using one microgram of total RNA using the SuperScript® III RT enzyme (Invitrogen, USA).

### *In-silico* identification of drought responsive genes

To predict drought responsive genes in pigeonpea, gene set annotated with pigeonpea genome assembly v5 (Varshney et al., 2012) was downloaded from International Initiative for Pigeonpea Genomics (IIPG: http://www.icrisat.org/gt-bt/iipg/Home.html). In addition, HMM profile of USP domain (PF00582) was retrieved from Pfam database (http://pfam.sanger.ac.uk/; Finn et al., 2010). The whole gene set was searched using “hmmsearch” program of HMMER 3.0 with HMM profile of the USP domain. Genes that detected sections encoding the USP domain, above the default inclusion threshold with statistically significant domain architecture were selected as USP domain-encoding genes.

Sequences were also subjected to protein homology search using BLASTP against Swiss-Prot and TrEMBL databases to further determine the identity of the selected genes containing USP domains. The identities obtained from the two databases were used in UniProtKB database to retrieve the protein names, location, biological pathways, and gene ontology identity with the help of an in-house Perl script. Gene ontology enrichment analysis was performed using the BiNGO tool with *p*-value cut-off of ≤ 0.05 (Maere et al., 2005).

### Primer designing and qRT-PCR

Gene specific primer pairs were designed from the exonic regions of the selected genes. Primer3 software (http://probes.pw.usda.gov/cgi-bin/batchprimer3/batchprimer3.cgi) was used for primer designing using the following criteria: annealing temperature (Tm) in the range of 55–60°C with an average of 57°C, amplicon size of 150–200 bp, primer length of 20 ± 5 bp and GC% of 50 ± 5 (Supplementary Table 1). All the designed primer pairs were custom synthesized by MWG (MWG-Biotech AG, Bangalore, India).

qRT-PCR was carried out using ABI SYBR® GREEN PCR reaction on an ABI Fast7500 System (Applied Biosystems, Foster City, CA, USA) following manufacturer's instructions. PCR conditions maintained for all qRT-PCR reactions were 2 min at 50°C, 10 min at 95°C, and 40 cycles of 15 s at 95°C and 1 min at 60°C. The melt curve analysis was conducted for all 51 primer pairs. Only after confirming the observed single peak with all the selected tissue samples, primers were used further for qRT-PCR analysis.

Each reaction was carried out in three biological and two technical replicates along with no template control. The differential expression values of drought responsive genes were normalized with *ACT1* as the reference gene (Supplementary Table 2). Statistical comparison between data obtained from three genotypes (ICPL 227, ICPL 8755 and ICPL 151) was performed using Tukey's *post-hoc* multiple comparison test using SPSS (version 16.0) whereas Student *t*-test was used to compare tolerant and susceptible genotypes of other four legume crops.

## Results

### Identification of drought responsive genes

The gene set consisting of 48,680 gene models (Varshney et al., 2012) was used to search the USP domain-encoding genes. As a result, 71 genes were found to encode USP domain, of which 51 were identified to be above the inclusion threshold with *E* < 0.01 (Supplementary Table 3). Of these genes, 49 also showed identity to the 111 drought responsive genes reported earlier in pigeonpea (Varshney et al., 2012).

### Functional classification of drought responsive genes

**Figure 1:**
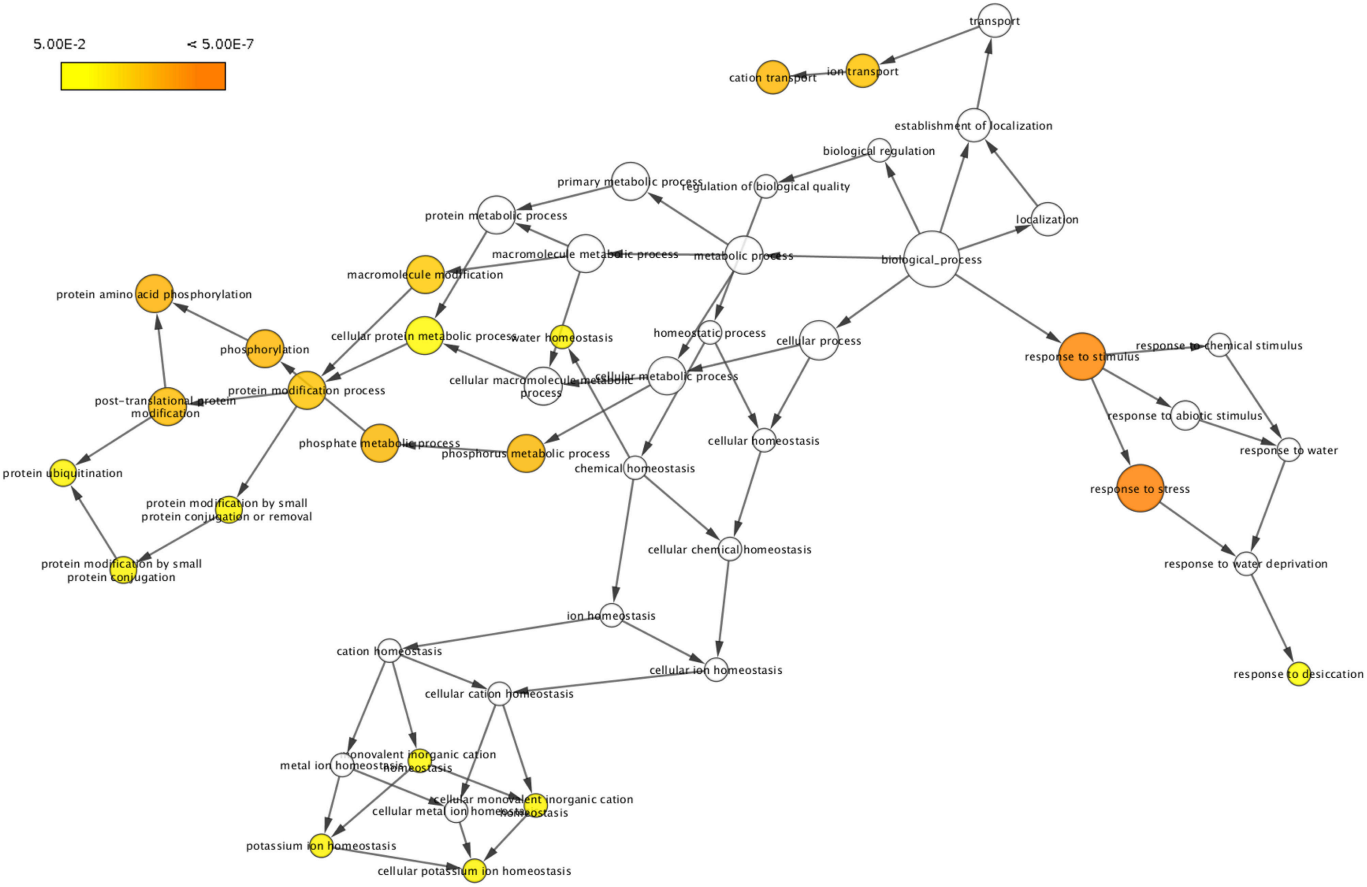
**Gene ontology (GO) enrichment of drought responsive genes**. Genes showing differential regulation were analyzed using BiNGO and the biological process terms showing significant enrichment are presented. The colors shades represent the following significance level; white-no significance difference; yellow *P* = 0.05, orange *P* < 0.0000005.

To classify the 51 drought responsive genes based on their functional annotations, BLASTP search was performed against Swiss-Prot and TrEMBL databases. This analysis revealed that about 25.5% of the genes were classified as related to molecular functions such as catalytic activity (8%), transporter activity (5%), and binding (6%), whereas 27.4% of the genes were found to be related to cellular component such as ubiquitin ligase complex (5%), membrane (6%), organelle (5%), membrane part (5%), plastid part (1%), and cell part (11%). The genes involved in biological processes formed 47% and included response to stress (13%), metabolic process (8%), cellular process (12%), homeostatic process (3%), single-organism process (6%), localization (5%) and establishment of localization (5%). Gene ontology (GO) term enrichment performed using BiNGO for these 51 genes as visualized in Cytoscape has been presented in Figure 1. Detailed information of the corresponding protein name, GO term and ontology identities of these genes has also been provided in the Supplementary Table 4 and Supplementary Figure 1.

On the basis of encoded proteins, the analyzed genes were further classified into six different groups namely, uncharacterized proteins (10 gene), universal stress protein A-(*uspA*) like protein (17 genes), plant U-box proteins (13 genes), cation/H(+) antiporter (*CHX*) proteins (6 genes), serine/threonine-protein kinase (4 genes), and probable nucleoredoxin (1 gene) (Supplementary Table 4).

### Differentially expressed drought responsive genes

**Figure 2:**
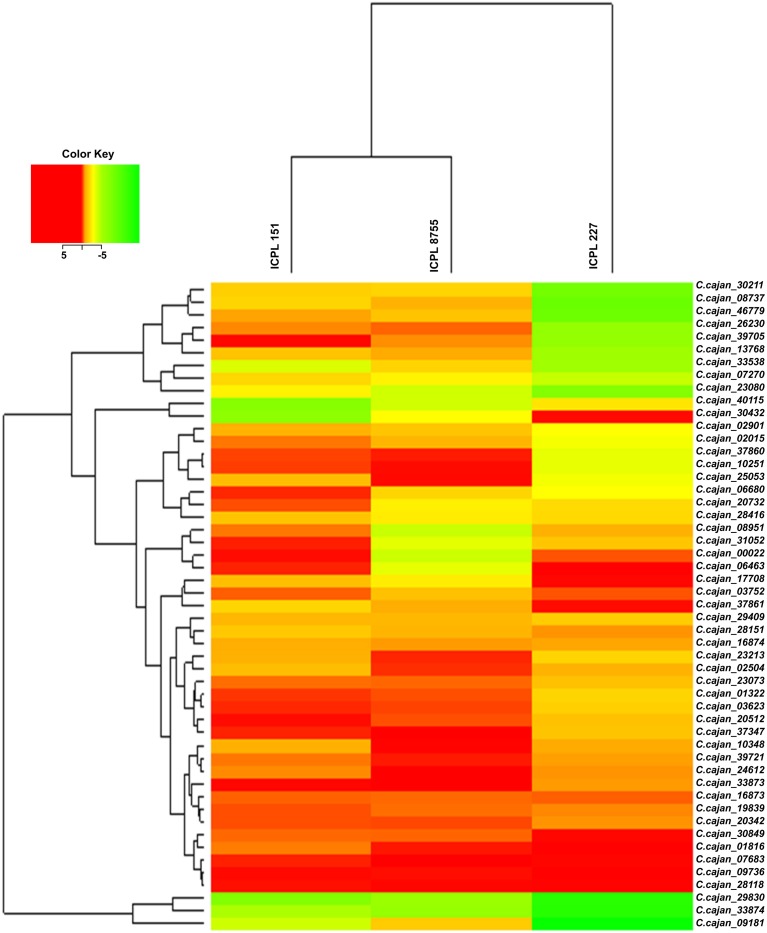
**Heat map of 51 drought responsive genes in MDT and LDT genotypes during drought stress condition**. Heat map depicting clusters of differentially expressed 51 drought responsive genes. Induced genes are represented in red and suppressed genes are represented in green. The color scale at the top right represents the log-transformed RPKM-value.

Gene specific primer pairs were designed from the exonic regions of 51 selected drought responsive genes for validation using qRT-PCR (Supplementary Table 3). Hierarchical cluster analysis of expression data of these genes showed a range of differential gene expression among three genotypes (ICPL 151, ICPL 8755, and ICPL 227) under stressed and controlled conditions. This analysis revealed that the less drought tolerant (LDT) genotypes, ICPL 151 and ICPL 8755 clustered separately from the more drought tolerant (MDT) genotype, ICPL 227 (Figure 2). Expression data was analyzed further in two different ways: (1) comparison between stressed and control samples for each genotype and (2) pair-wise comparison between genotypes. Expression analysis for each genotype with respect to stressed and control samples identified 18 genes in ICPL 227, six genes in ICPL 151 and eight genes in ICPL 8755 with significant expression variation. Genes with more than two-fold expression difference in each of these genotypes have been listed in Table 2. However, the expression analysis among the genotype pairs, ICPL 227 with ICPL 8755 (Supplementary Table 5) and ICPL 227 with ICPL 151 (Supplementary Table 6) has identified 11 genes in each case.

**Table 2:** **List of common genes with more than two-fold difference between more and less drought tolerant genotypes**.

**Gene-Id**	**ICPL 227 (More tolerant)**	**ICPL 151 (Less tolerant)**	**ICPL 8755 (Less tolerant)**	**Uniprot-Id**	**Protein name**
*C.cajan_26230*	5.14	−0.20	−0.40	Q9SW11	U-box domain-containing protein 35
*C.cajan_39705*	5.19	−0.95	−0.16	Q9SW11	U-box domain-containing protein 35
*C.cajan_09181*	13.50	3.16	0.80	Q8GZ84	U-box domain-containing protein 36
*C.cajan_30211*	7.13	0.86	1.00	Q9FKG6	U-box domain-containing protein 52
*C.cajan_46779*	7.47	−0.03	0.60	Q9SIT5	Cation/H(+) antiporter 15
*C.cajan_08737*	7.70	1.03	0.24	I1JEJ0	Uncharacterized protein
*C.cajan_13768*	4.56	0.58	0.04	Q8LGG8	Universal stress protein A-like protein
*C.cajan_23080*	6.19	1.70	3.10	Q57951	Universal stress protein
*C.cajan_29830*	11.40	6.30	4.78	Q8LGG8	Universal stress protein A-like protein
*C.cajan_33874*	11.68	3.98	5.03	Q8LGG8	Universal stress protein A-like protein

**Figure 3:**
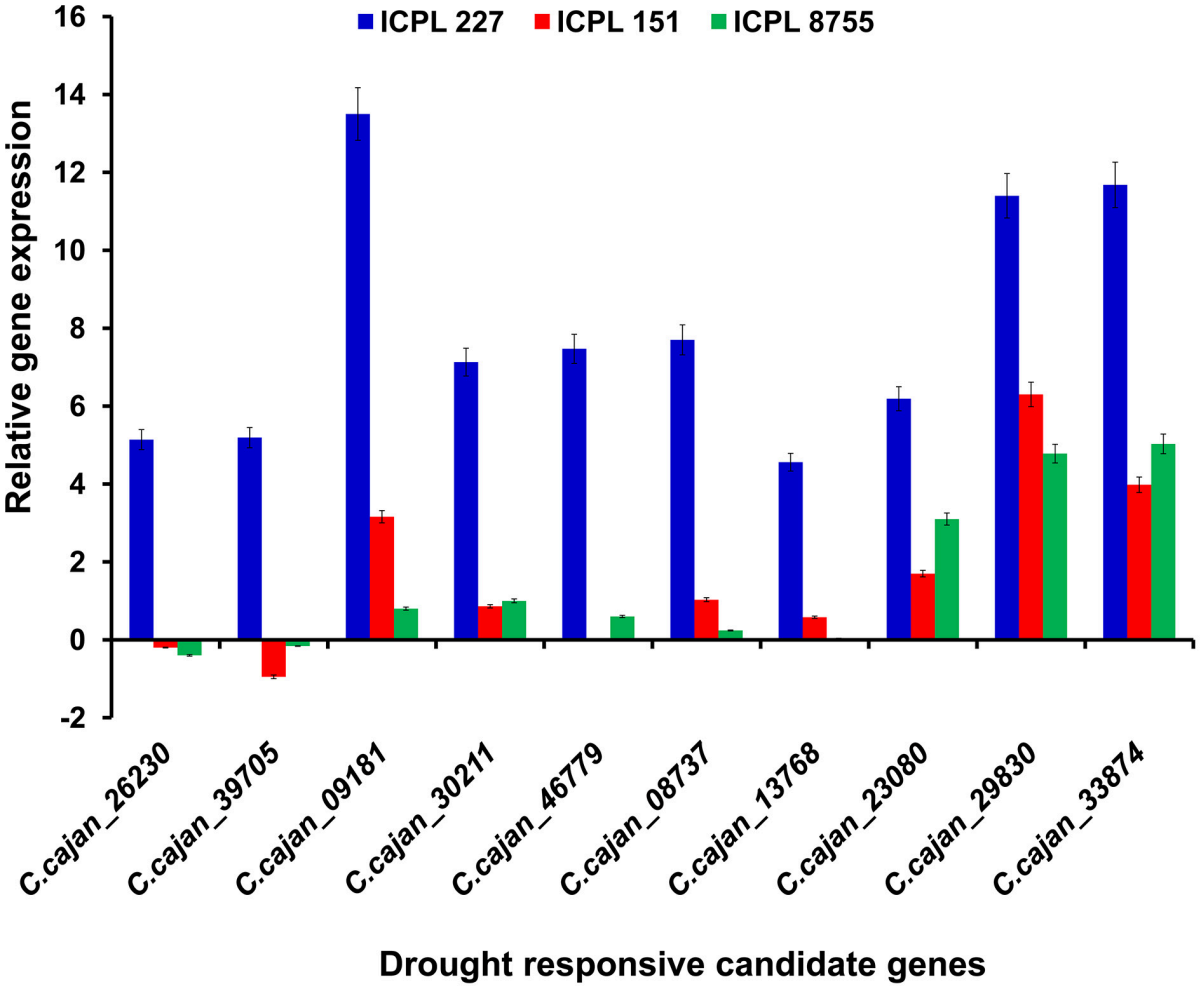
**Graph showing the relative transcript abundance for 10 genes in three genotypes, namely ICPL 227 (MDT genotype), ICPL 151, and ICPL 8755 (LDT genotypes)**. The y-axis represents the relative transcript abundance for the drought responsive genes depicted on the x-axis. The graph clearly shows ≥2-fold up-regulation of two genes namely, *C.cajan_29830* and *C.cajan_33874* in all the three genotypes with varying drought tolerance.

Furthermore, the relative transcript abundance was compared between the MDT genotype, ICPL 227 and the LDT genotypes, ICPL 8755 and ICPL 151 to identify the common genes. As a result, among these genotypes, 10 genes were found to be common and showed large differences in the relative transcript abundance (Table 2 and Figure 3). These genes encodes four different classes of proteins, which include plant U-box proteins (four genes), cation/H(+) antiporter (*CHX*) proteins (one gene), uncharacterized proteins (one gene), and universal stress protein A-(*uspA*) like protein (four genes). Four genes encoding plant U-box proteins namely, *C.cajan_26230, C.cajan_39705, C.cajan_09181*, and *C.cajan_30211* showed significant up-regulation in ICPL 227 in comparison to ICPL 151 and ICPL 8755. The gene, *C.cajan_26230* showed 5.14-fold expression variation in ICPL 227 in comparison to ICPL 151 (–0.20-fold) and ICPL 8755 (–0.40-fold). Similarly, the gene *C.cajan_39705* showed higher level of expression (5.19-fold) in ICPL 227 as compared to ICPL 151 (–0.95-fold) and ICPL 8755 (–0.16-fold), whereas *C.cajan_09181* showed 13.5-fold gene expression variations in ICPL 227 compared to 3.16-fold in ICPL 151 and 0.80-fold in ICPL 8755. Likewise, for *C.cajan_30211*, the expression difference observed in ICPL 227 was high (7.13) as compared to that in ICPL 151 (0.86) and ICPL 8755 (1.00).

In the case of *CHX* gene, *C.cajan_46779* showed significant up-regulation in ICPL 227 (7.47-fold) unlike ICPL 151 (−0.03-fold) and ICPL 8755 (0.6-fold). Also, expression profiling of the gene, *C.cajan_08737* encoding uncharacterized protein revealed 7.70-folds relative expression variation in ICPL 227 as compared to ICPL 151 (1.03) and ICPL 8755 (0.24). Four genes namely, *C.cajan_13768, C.cajan_23080, C.cajan_29830*, and *C.cajan_33874* encoding universal stress protein showed large relative transcript abundance differences among three genotypes. The gene, *C.cajan_13768* showed 4.56-fold relative expression variation in ICPL 227 as compared to ICPL 151 (0.58-fold) and ICPL 8755 (0.04-fold). Another gene *C.cajan_23080* was having 6.19-fold relative expression in ICPL 227 as compared to ICPL151 (1.70-fold) and ICPL 8755 (3.10-fold). Interestingly, two genes showed 11.40, 6.30, 4.78 (*C.cajan_29830*) and 11.68, 3.98, 5.03 (*C.cajan_33874*) folds up-regulation in ICPL 227, ICPL 151, and ICPL 8755, respectively (Figure 3).

### Comparative expression profiling of candidate genes across legumes

**Figure 4:**
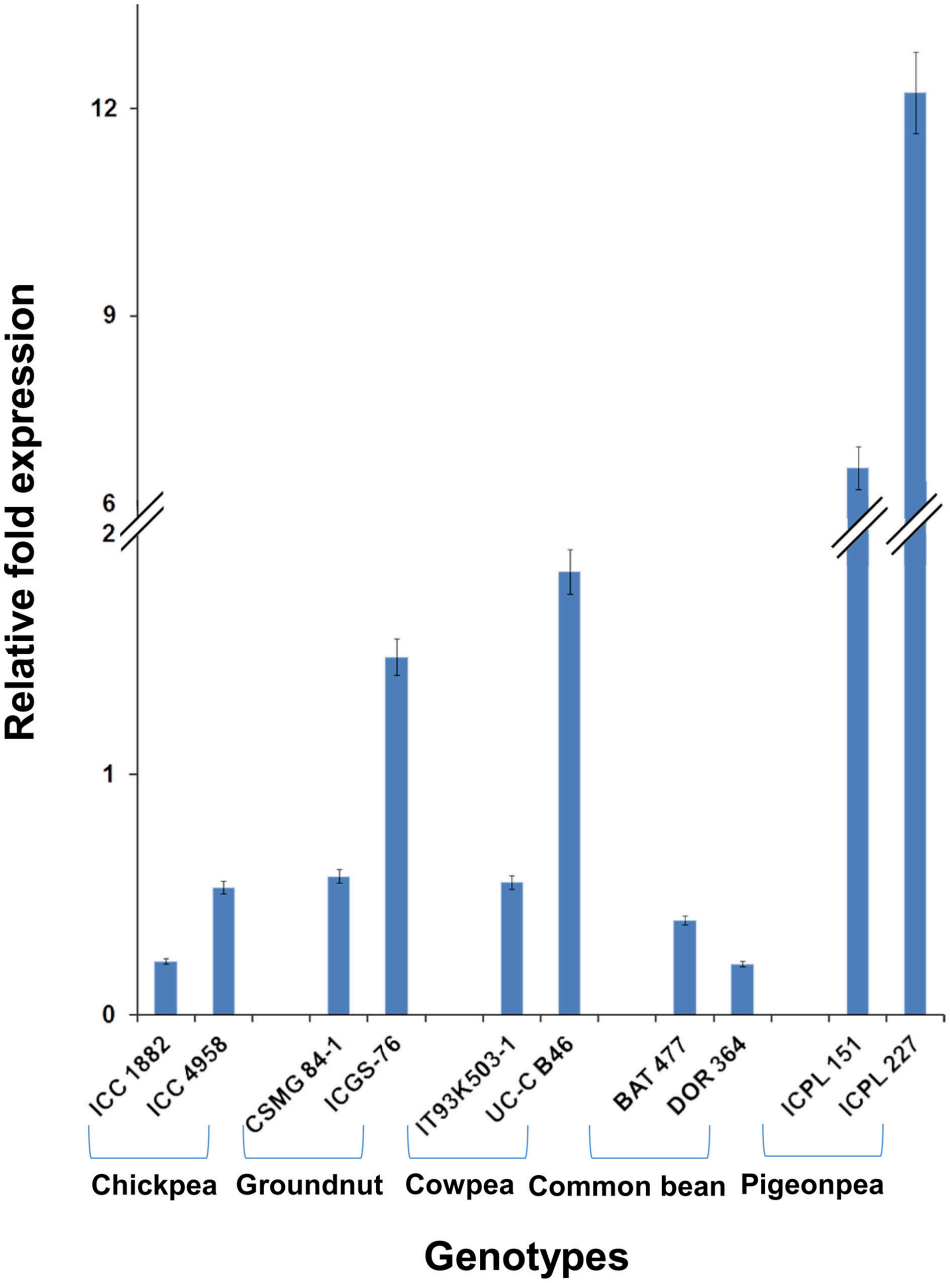
**Expression analysis of identified drought responsive gene, *C.cajan_29830* in the root tissues of tolerant and susceptible genotypes of the four legumes**. The expression analysis was carried out using chickpea genotypes, ICC 4958 (tolerant) and ICC 1882 (susceptible), CSMG 84-1 (tolerant) and ICGS-76 (susceptible) for groundnut, IT93K503-1 (tolerant) and UC-C B46 (susceptible) for cowpea, and BAT 477 (tolerant) and DOR 364 (susceptible) for common bean, in comparison to the pigeonpea genotypes, ICPL 151 (LDT genotype) and ICPL 227 (MDT genotype).

**Figure 5:**
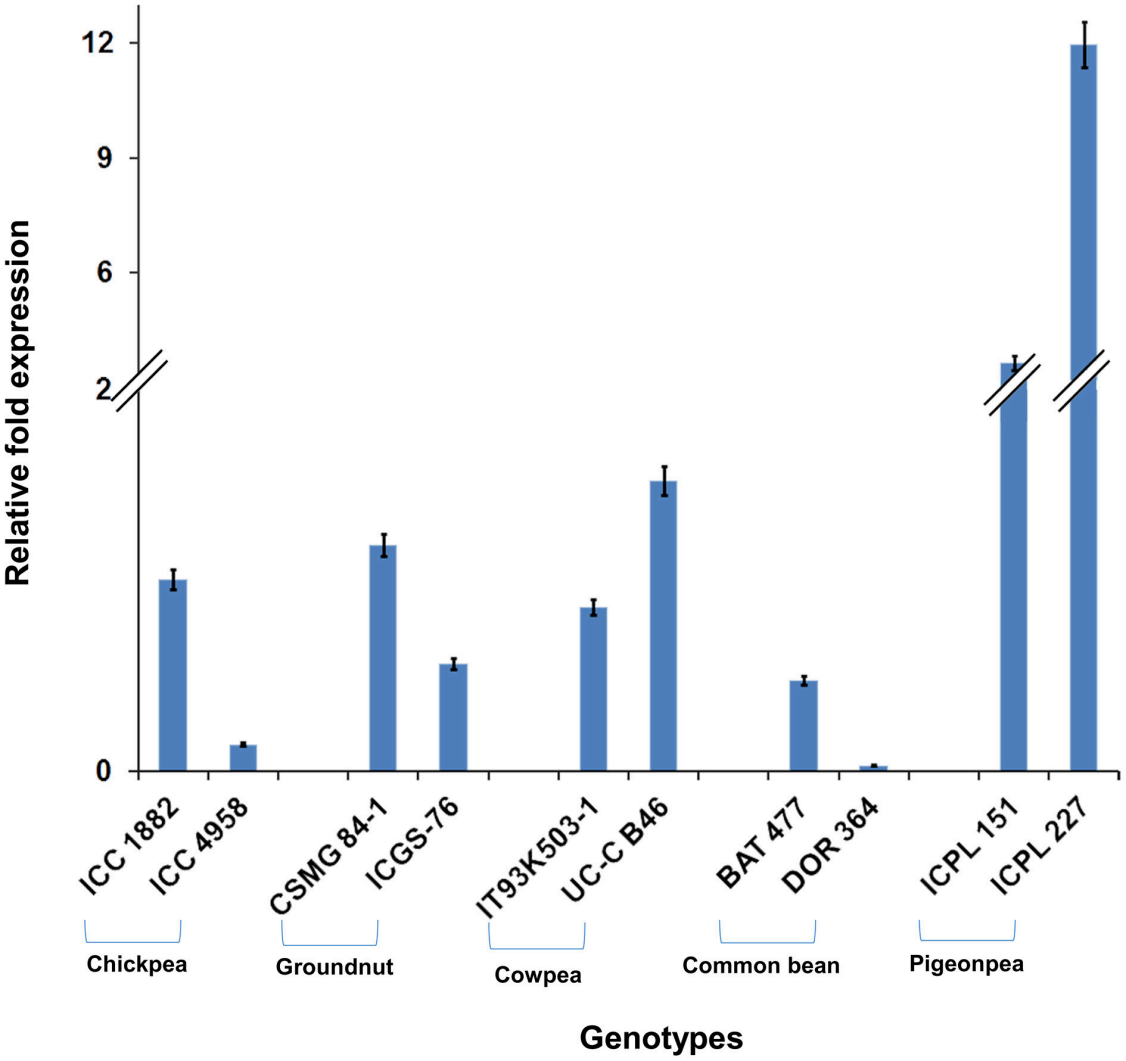
**Expression analysis of identified drought responsive gene, *C.cajan_33874* in the root tissues of tolerant and susceptible genotypes of the four legumes**. The expression analysis was carried out using chickpea genotypes, ICC 4958 (tolerant) and ICC 1882 (susceptible), CSMG 84-1 (tolerant) and ICGS-76 (susceptible) for groundnut, IT93K503-1 (tolerant) and UC-C B46 (susceptible) for cowpea, and BAT 477 (tolerant) and DOR 364 (susceptible) for common bean, in comparison to the pigeonpea genotypes, ICPL 151 (LDT genotype) and ICPL 227 (MDT genotype).

Among the 10 common differentially expressed genes among MDT and LDT genotypes, two genes namely, *C.cajan_29830* and *C.cajan_33874* showed marked up-regulation in all the three drought tolerant genotypes (Supplementary Figure 2). These two genes were further selected for validation using tolerant and susceptible genotypes in chickpea, groundnut, common bean, and cowpea. For gene normalization, glyceraldehyde 3-phosphate dehydrogenase (*GAPDH*) for chickpea (Garg et al., 2010), alcohol dehydrogenase (*ADH*) for groundnut (Reddy et al., 2013) while β-tubulin for common bean and cowpea (Eticha et al., 2010) were used as internal control (Supplementary Table 2 and Supplementary Figure 3). To perform normalization, the reference genes which were reported to be stable for each of the legume crops were considered. In terms of expression profiling, in chickpea, ICC 4958 (tolerant genotype), showed 0.53, 0.14, and ICC 1882 (susceptible genotype), showed 0.23-, 1.01-fold differential gene expressions for *C.cajan_29830* (Figure 4) and *C.cajan_33874* (Figure 5), respectively. Similarly in groundnut, these two genes showed an expression variation of 0.58, 1.19 in CSMG 84-1 (tolerant genotype) for *C.cajan_29830* and 1.49, 0.56 in ICGS-76 (susceptible genotype) for *C.cajan_33874*. In the case of cowpea, the tolerant genotype (IT93K503-1) showed 0.55, 0.86 while the susceptible genotype (UC-C B46), showed 1.84-, 1.52-fold expression difference for the two genes, *C.cajan_29830* and *C.cajan_33874*, respectively. Similarly, in common bean, BAT 477 (tolerant genotype), showed 0.21, 0.03, and DOR 364 (susceptible genotype) showed 0.39, 0.48 differential gene expression for *C.cajan_29830* and *C.cajan_33874*, respectively. Overall, the gene expression variation between the tolerant and susceptible genotypes of the four legumes for the selected genes was less than 2-folds. Thus, the present study implies that these two genes might specifically be involved in contributing drought tolerance in pigeonpea.

## Discussion

This study has utilized genome sequence information for selecting genes encoding proteins containing USP domain and were validated for their role in drought tolerance in pigeonpea using qRT-PCR. Genes encoding protein with USP domain are known to be involved in a myriad of stress responses and any mutation in these genes may cause loss of efficacy in combating stresses (Drumm et al., 2009; Isokpehi et al., 2011; Shokry et al., 2014). USP domain has been found to be evolutionary conserved in a number of crop species such as cassava, soybean, finger millet, and peanut (Govind et al., 2009; Deeplanaik et al., 2013; Turyagyenda et al., 2013).

Earlier studies have provided evidences that stress-responsive genes encoding proteins with USP domain are useful in stress signal perception and subsequently lead to functionally efficient proteins. These proteins have been found to be involved in protecting cellular structures and cell molecules under stress conditions (Waditee et al., 2002; Majee et al., 2004; Dastidar et al., 2006; Govind et al., 2009). For instance, under water deficit conditions, out of 50 genes selected in peanut, only *HSP70* gene showed association with drought stress response (Govind et al., 2009). Similarly, 10 genes conferring drought tolerance were characterized in cassava (Turyagyenda et al., 2013). In the case of pigeonpea, homology search provided 71 genes encoding USP domain, of which 51 genes with pure domain architecture were selected for further validation in pigeonpea. During water stress conditions, plant responds at both cellular as well as molecular level by accumulating osmolytes and proteins involved in stress response and/or tolerance (Yamaguchi-Shinozaki and Shinozaki, 2006). Stress response at cellular level such as cell proliferation, differentiation, stomatal closure, repression of cell growth generally lead to induced expression of drought responsive genes (Yamaguchi-Shinozaki and Shinozaki, 2005). Among the selected 51 genes, majority of the genes were found to be related to response to stress followed by cellular processes.

The three pigeonpea genotypes selected for expression profiling in the present study exhibited varying degree of tolerance to drought stress (Lopez et al., 1996; Saxena et al., 2011). The expression variation of candidate genes in stressed tissues can be compared with well water controls at specific time frame (VanGuilder et al., 2008) using qRT-PCR. A set of 10 genes, which were identified in the MDT and LDT genotypes showed large difference in the relative transcript abundance. These genes were found to be related to plant U-box proteins, cation/H(+) antiporter (*CHX*) proteins, uncharacterized protein and universal stress protein A-(*uspA*) like protein.

Plant U-box E3 ligases have been found to be involved in enhanced drought, salinity, cold and heat tolerance in *Arabidopsis thaliana* (Lyzenga and Stone, 2011). Whereas, Ubiquitin-protein ligases (E3s) determine the substrate specificity of ubiquitylation and plays an important role in protein post-translational modification in higher plants (Liu and Walters, 2010). Based on the structure, Ubiquitin-protein ligases (E3s) has been classified into two families, the HECT and RING-finger (U-Box) families (Hatakeyama and Nakayama, 2003). Molecular and cellular characterization of U-Box protein-coding genes in hot pepper (Cho et al., 2006) and *Arabidopsis* (Cho et al., 2008) has clearly demonstrated the role of U-Box protein-coding genes in drought tolerance. The four plant U-Box protein-coding genes (*C.cajan_26230, C.cajan_39705, C.cajan_09181*, and *C.cajan_30211*) identified between the MDT and LDT genotypes showed significant differences in the relative transcript abundance.

One gene (*C.cajan_46779*) belonging to cation/H(+) exchanger (*CHX*) group was also found to be differentially expressed between the two genotypes studied. In *Arabidopsis, CHX* gene family was found to play an important role in osmotic adjustment and K+ homeostasis (Sze et al., 2004). Therefore, the finding suggests that the gene, *C.cajan_46779* belonging to *CHX* gene family may also be playing an important role during drought stress condition in pigeonpea. Another gene, *C.cajan_08737*, annotated as uncharacterized protein, was also found to be differentially expressed among MDT and LDT genotypes suggesting its role in drought tolerance. Another class of genes (*C.cajan_13768, C.cajan_23080, C.cajan_29830*, and *C.cajan_33874*) having differences in the relative transcript abundance between MDT and LDT genotypes was found to have the *uspA* domain. The *uspA* domain is also known to play a vital role in survival during cellular growth arrest. Genes belonging to this domain help in oxidative stress resistance and initiates defense against superoxide-generating agents (Nachin et al., 2005).

Pigeonpea is one of the most drought tolerant legume crops (Varshney et al., 2009). Therefore, the expression variation of the two differentially expressed genes, *C.cajan_29830* and *C.cajan_33874* identified across all the three pigeonpea genotypes was studied across four other legumes. For these two genes (*C.cajan_29830* and *C.cajan_33874*), chickpea (78.5 and 82%), groundnut (77.6 and 79.5%) and common bean (85.9 and 86.4%) genes showed high sequence identity with pigeonpea, respectively. Based on this observation, two genes encoding Universal stress protein A-like protein seem to be highly conserved among the legumes studied. However, in the four legume crops, namely chickpea, groundnut, common bean, and cowpea, these two genes did not show any expression variation between the drought tolerant and susceptible genotypes. This may also be due to different strategies acquired by different legumes for drought adaptation mechanism (Mir et al., 2012). This study also showed the involvement of U-Box protein-coding in having some specific role in drought tolerance mechanisms in pigeonpea. Many such conserved U-Box protein-coding genes were found to be over-expressed in different plant species to enhance drought tolerance (Lyzenga and Stone, 2011).

Thus, expression analysis of the 51 drought responsive genes has provided a set of 10 genes belongs to plant U-Box proteins, cation/H(+) antiporter (*CHX*) proteins, uncharacterized proteins and universal stress protein A-(*uspA*) like protein. This candidate gene-based approach could provide useful insights into the molecular mechanisms involved in drought tolerance in pigeonpea. Moreover, the identified genes can also be validated at sequence level in different genetic backgrounds to detect the presence of sequence variations for the development of gene-based marker(s) for crop improvement and development of more tolerant breeding lines/hybrids through genomics-assisted breeding.

### Conflict of interest statement

The authors declare that the research was conducted in the absence of any commercial or financial relationships that could be construed as a potential conflict of interest.

## Supplementary material

Supplementary Figure 1 **Functional classification of the 51 drought responsive pigeonpea genes having USP domains**. These categories include (a) 25.5% in molecular function: catalytic activity (8%), transporter activity (5%) and binding (6%), (b) 27.4% in cellular component: ubiquitin ligase complex (5%), membrane (6%), organelle (5%), membrane part (5%), plastid part (1%) and cell part (11%), and (c) 47% in biological process: response to stress (13%), metabolic process (8%), cellular process (12%), homeostatic process (3%), single-organism process (6%), localization (5%), and establishment of localization (5%).

Supplementary Figure 2 **Expression variation of two candidate genes (*C.cajan_29830* and *C.cajan_33874*) between MDT and LDT genotypes**. Differentially expressed genes were identified with ≥2-fold expression variation across the three pigeonpea genotypes, namely ICPL 227 (MDT genotype), ICPL 151, and ICPL 8755 (LDT genotypes). The different letters above the bars were considered as statistically significant between each other.

Supplementary Figure 3 **Cross generic amplification check. (A)** The melt curve obtained for the two qRT-PCR primer sets, namely *C.cajan_29830* (Top) and *C.cajan_33874* (Bottom) in the resistant and the susceptible genotypes of the four legumes studied. A: ICC 4958; B: ICC 1882; C: IT93K503-1; D: CSMG 84-1, E: BAT 477; F: UC-C B46; G: DOR 364 and H: ICGS-76. **(B)** 2% agarose gel showing the amplification of corresponding genes in the resistant and the susceptible genotypes of the four legumes studied to the pigeonpea genes (*C.cajan_29830*-Top and *C.cajan_33874*-Bottom).

Supplementary Table 1 **List of primer pairs used for qRT-PCR analysis**.

Supplementary Table 2 **List of housekeeping genes used for qRT-PCR analysis**.

Supplementary Table 3 **HMM Search output for USP domain in pigeonpea genes set**.

Supplementary Table 4 **Protein names, gene ontology terms (GO_term) and ontology identities (GO_ID) of 51 drought responsive genes**.

Supplementary Table 5 **List of genes showing more than two-fold difference between ICPL 227 and ICPL 8755**.

Supplementary Table 6 **List of genes showing more than two-fold difference between ICPL 227 and ICPL 151**.
