# Supplementary figures and images for: Identification and Validation of Selected Universal Stress Protein Domain Containing Drought-Responsive Genes in Pigeonpea (Cajanus cajan L.)
Source: Front Plant Sci. 2016 Jan 6;6:1065. doi: 10.3389/fpls.2015.01065 (PMC4701917; doi:10.3389/fpls.2015.01065)

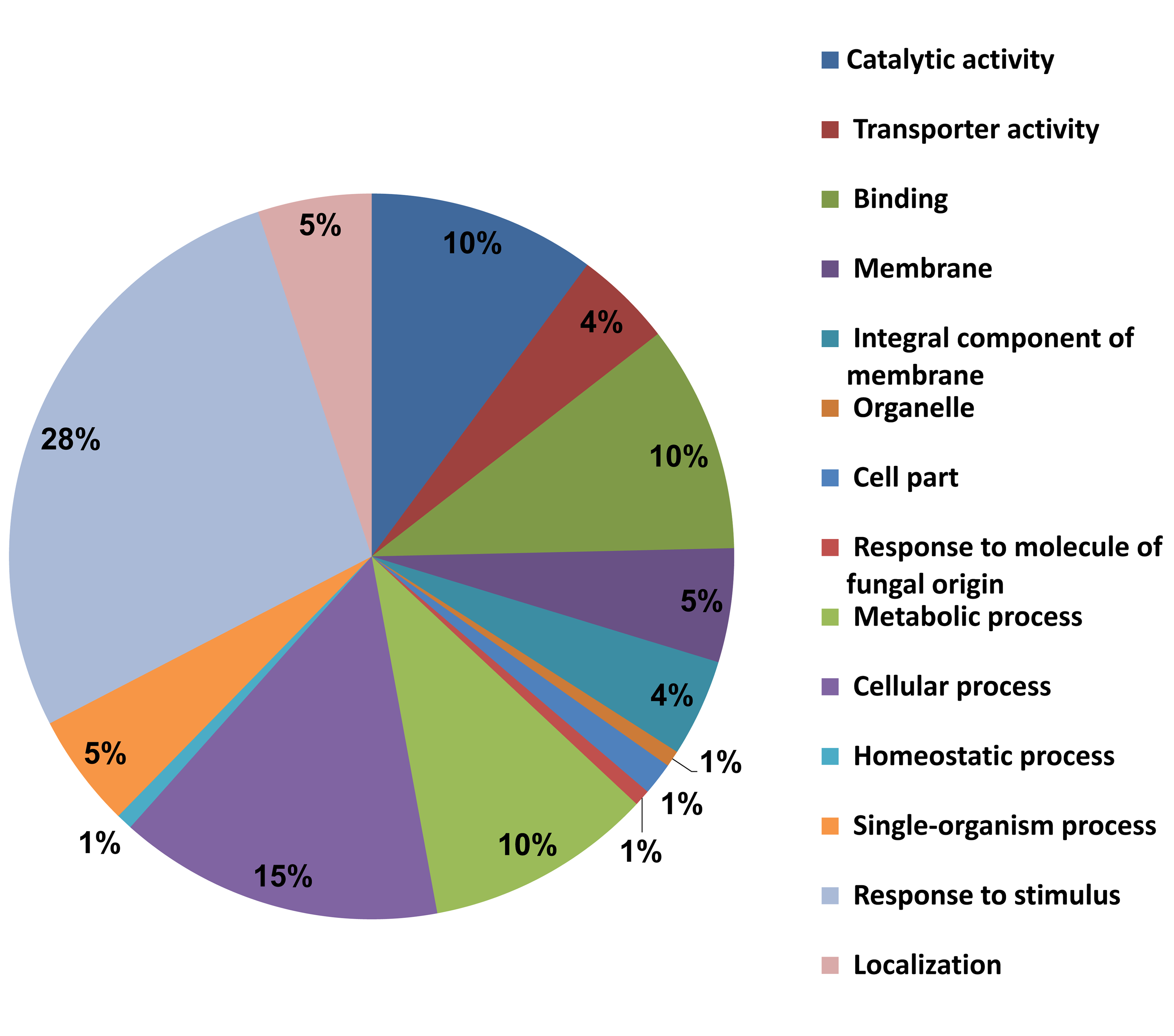

Supplement: Supplementary Figure 1 — Functional classification of the 51 drought responsive pigeonpea genes having USP domains. These categories include (a) 25.5% in molecular function: catalytic activity (8%), transporter activity (5%) and binding (6%), (b) 27.4% in cellular component: ubiquitin ligase complex (5%), membrane (6%), organelle (5%), membrane part (5%), plastid part (1%) and cell part (11%), and (c) 47% in biological process: response to stress (13%), metabolic process (8%), cellular process (12%), homeostatic process (3%), single-organism process (6%), localization (5%), and establishment of localization (5%). [file Image1.TIF]

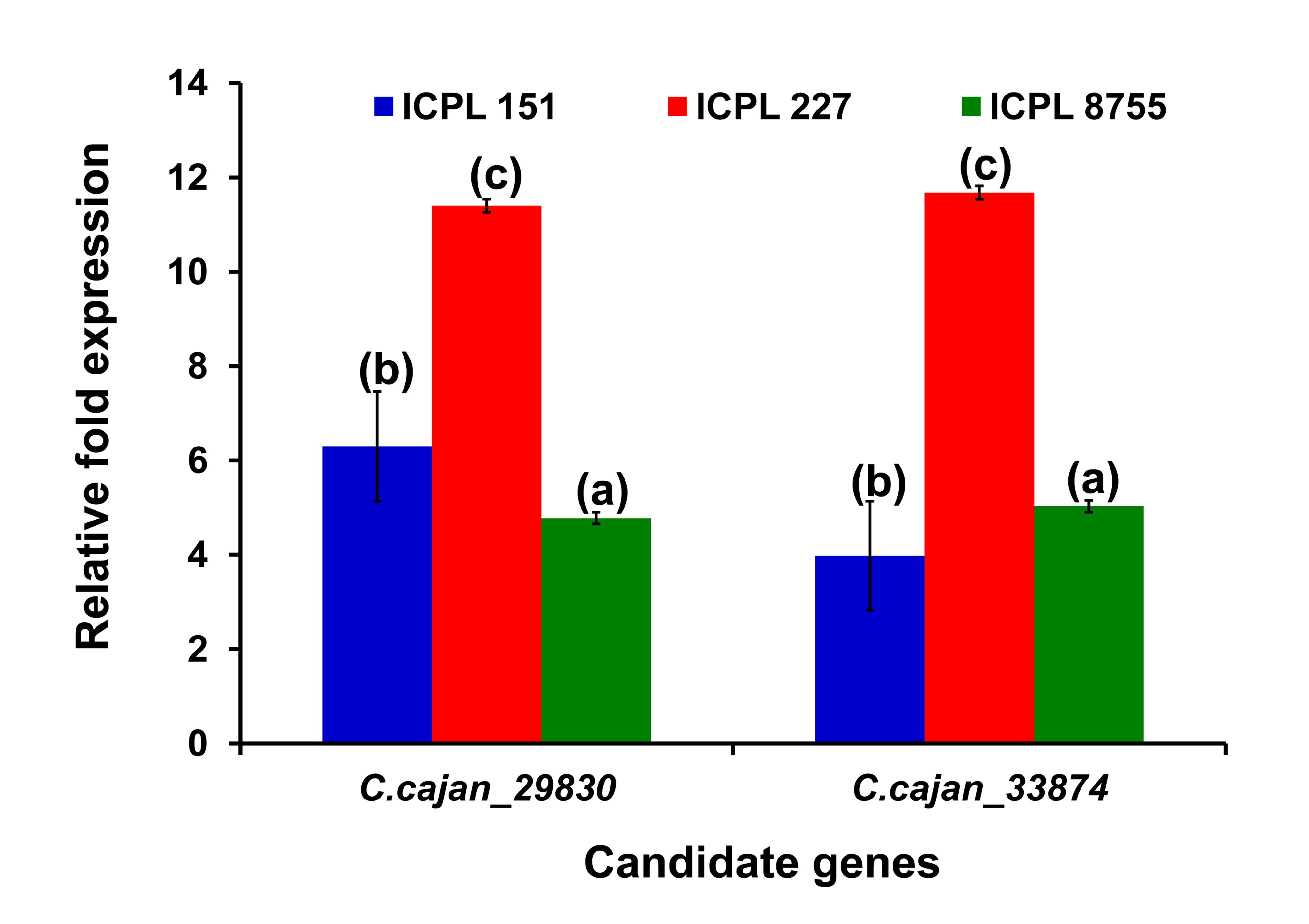

Supplement: Supplementary Figure 2 — Expression variation of two candidate genes (C.cajan_29830 and C.cajan_33874) between MDT and LDT genotypes. Differentially expressed genes were identified with ≥2-fold expression variation across the three pigeonpea genotypes, namely ICPL 227 (MDT genotype), ICPL 151, and ICPL 8755 (LDT genotypes). The different letters above the bars were considered as statistically significant between each other. [file Image2.TIF]

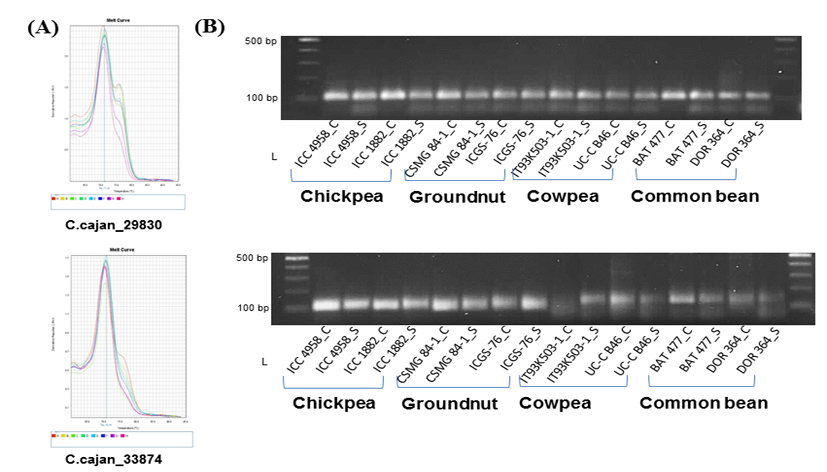

Supplement: Supplementary Figure 3 — Cross generic amplification check. (A) The melt curve obtained for the two qRT-PCR primer sets, namely C.cajan_29830 (Top) and C.cajan_33874 (Bottom) in the resistant and the susceptible genotypes of the four legumes studied. A: ICC 4958; B: ICC 1882; C: IT93K503-1; D: CSMG 84-1, E: BAT 477; F: UC-C B46; G: DOR 364 and H: ICGS-76. (B) 2% agarose gel showing the amplification of corresponding genes in the resistant and the susceptible genotypes of the four legumes studied to the pigeonpea genes (C.cajan_29830-Top and C.cajan_33874-Bottom). [file Image3.tif]
