# Supplementary material for: Identification and Validation of Selected Universal Stress Protein Domain Containing Drought-Responsive Genes in Pigeonpea (Cajanus cajan L.)
Source: Front Plant Sci. 2016 Jan 6;6:1065. doi: 10.3389/fpls.2015.01065 (PMC4701917; doi:10.3389/fpls.2015.01065)
Supplement: Supplementary Table 1 — List of primer pairs used for qRT-PCR analysis. [file Table1.DOCX]

**Supplementary Table 1.** List of primer pairs used for qRT-PCR analysis

| **S. No.** | **Gene name** | **Primer_id** | **Primer sequence** | **Expected size (bp)** |  |
| --- | --- | --- | --- | --- | --- |
| 1 | | *C.cajan_37347* | Cc_cds_37347_F | GAGGATCTTGTGTCTGGT | 161 |
|  | |  | Cc_cds_37347_R | AGAGAAACAGCCAGAGTC |  |
| 2 | | *C.cajan_23073* | Cc_cds_23073_F | AGTGAGACCTATGGTTGG | 180 |
|  | |  | Cc_cds_23073_R | GTTATCATCCCACTCCTC |  |
| 3 | | *C.cajan_03623* | Cc_cds_03623_F | GAGACCTGTTGATTCCTC | 165 |
|  | |  | Cc_cds_03623_R | GCAGTTTGTCTCACACAG |  |
| 4 | | *C.cajan_19839* | Cc_cds_19839_F | CTCACCCTTTTCTCACTC | 179 |
|  | |  | Cc_cds_19839_R | GATGCTGGAACCATCTAC |  |
| 5 | | *C.cajan_03752* | Cc_cds_03752_F | GACCCAGAGTATCAGAGAAC | 172 |
|  | |  | Cc_cds_03752_R | CTCCAGCTGATGTGTCTA |  |
| 6 | | *C.cajan_10348* | Cc_cds_10348_F | TAGAGATGGATCCACGAG | 187 |
|  | |  | Cc_cds_10348_R | GGTGAGTCTGTGTTAGTGAG |  |
| 7 | | *C.cajan_20732* | Cc_cds_20732_F | GTCTCAGTGTGTTCTGACTC | 173 |
|  | |  | Cc_cds_20732_R | TCGTAGACAGCATCATCC |  |
| 8 | | *C.cajan_09736* | Cc_cds_09736_F | AGCTTCTCCACTGCTAAC | 187 |
|  | |  | Cc_cds_09736_R | GACCCTTCCCAACTACTA |  |
| 9 | | *C.cajan_28416* | Cc_cds_28416_F | TCTCCAAGAGTAGCAAGC | 174 |
|  | |  | Cc_cds_28416_R | TCTCAGGCTCTCTGAACT |  |
| 10 | | *C.cajan_37861* | Cc_cds_37861_F | CAGCTCTGGGAAACTACT | 180 |
|  | |  | Cc_cds_37861_R | CTTATCACGAGCATCTCC |  |
| 11 | | *C.cajan_29830* | Cc_cds_29830_F | CTTCCACGTTCAATCTCC | 195 |
|  | |  | Cc_cds_29830_R | GATCTGACCTTACTGGTGAC |  |
| 12 | | *C.cajan_40115* | Cc_cds_40115_F | GAGTACCAGCAGACAGGTAT | 170 |
|  | |  | Cc_cds_40115_R | CAGTCAGTGAGTGATGGA |  |
| 13 | | *C.cajan_31052* | Cc_cds_31052_F | GAAGAGGTGACAAGATGG | 163 |
|  | |  | Cc_cds_31052_R | TACTCTGCTCAGAGCTACC |  |
| 14 | | *C.cajan_08951* | Cc_cds_08951_F | GTTGGCCTCTACTACACAG | 184 |
|  | |  | Cc_cds_08951_R | CTATCTAAAGCCGCAGGT |  |
| 15 | | *C.cajan_07683* | Cc_cds_07683_F | AACTACAGGGAAGTGACG | 172 |
|  | |  | Cc_cds_07683 | CAGCGAGTCTAGAAAGGT |  |
| 16 | | *C.cajan_06680* | Cc_cds_06680_F | GTCTTCAGAGAAGCAAGC | 188 |
|  | |  | Cc_cds_06680_R | CAATAGGCAAGACCTCAG |  |
| 17 | | *C.cajan_33874* | Cc_cds_33874_F | GAGTTTCGTGAGAAGGAG | 188 |
|  | |  | Cc_cds_33874_R | CTACCCATGACCAAAGAG |  |
| 18 | | *C.cajan_29409* | Cc_cds_29409_F | GTAGCTGTGGACTTCTCTG | 156 |
|  | |  | Cc_cds_29409_R | CAAAGGTGAACCAGTGTC |  |
| 19 | | *C.cajan_24612* | Cc_cds_24612_F | AAGAGAAGACTGCGAGAG | 167 |
|  | |  | Cc_cds_24612_R | GGAAAACGTGTAGGAGAC |  |
| 20 | | *C.cajan_26230* | Cc_cds_26230_F | GGAGATACGGTATAGAGGAG | 194 |
|  | |  | Cc_cds_26230_R | CATGCAGCTCAGTATGTC |  |
| 21 | | *C.cajan_02504* | Cc_cds_02504_F | CCTTCTGCTTTCTACACC | 180 |
|  | |  | Cc_cds_02504_R | GGCATCTCCTATACCAAC |  |
| 22 | | *C.cajan_39705* | Cc_cds_39705_F | CCAGATTACTGCTCTGTG | 177 |
|  | |  | Cc_cds_39705_R | CCTACTTCTAGGTGGTTGTC |  |
| 23 | | *C.cajan_28118* | Cc_cds_28118_F | GTGACTCGGATAATGGTG | 167 |
|  | |  | Cc_cds_28118_R | CCATCTTCATCTGGGACT |  |
| 24 | | *C.cajan_23080* | Cc_cds_23080_F | CTAGTGGAAGTGGTCGAA | 182 |
|  | |  | Cc_cds_23080_R | GGCCTCTTCACAATCATC |  |
| 25 | | *C.cajan_06463* | Cc_cds_06463_F | CTGTAGTGGTGGATTCTG | 184 |
|  | |  | Cc_cds_06463_R | CTACCCATGACTAGGAGACT |  |
| 26 | | *C.cajan_00022* | Cc_cds_00022_F | GCTTCTTCTAGGAGCTTG | 177 |
|  | |  | Cc_cds_00022_R | GTCTGATGGAGGAACAGT |  |
| 27 | | *C.cajan_09181* | Cc_cds_09181_F | CGCAGCAAGTGAGACTATAC | 158 |
|  | |  | Cc_cds_09181_R | AATAGTGGTGAGGTCCAG |  |
| 28 | | *C.cajan_25053* | Cc_cds_25053_F | CTACTGTTTCCGCTTCTG | 173 |
|  | |  | Cc_cds_25053_R | TCTAGGATCACCTTCCAC |  |
| 29 | | *C.cajan_16874* | Cc_cds_16874_F | ACCTCCAACTATGCTCTG | 175 |
|  | |  | Cc_cds_16874_R | GCTATGCTACTCCCTTTC |  |
| 30 | | *C.cajan_16873* | Cc_cds_16873_F | ATCTGAAGACTGCGAGAG | 158 |
|  | |  | Cc_cds_16873_R | TGAGAGAGACAGTGGTAGAG |  |
| 31 | | *C.cajan_23213* | Cc_cds_23213_F | CCAGTCTACAGGTCTGAAC | 171 |
|  | |  | Cc_cds_23213_R | CACTAAGCAGCCAAACTC |  |
| 32 | | *C.cajan_33538* | Cc_cds_33538_F | CCCTTATTCTCCTCTACG | 189 |
|  | |  | Cc_cds_33538_R | CACTCTCTACCCTCGTCT |  |
| 33 | | *C.cajan_07270* | Cc_cds_07270_F | GTTTACAAGGGACTGCTC | 168 |
|  | |  | Cc_cds_07270 | CACAAGTGACCTAGACTCTG |  |
| 34 | | *C.cajan_13768* | Cc_cds_13768_F | CTTCTTCTGTGCAGGTTG | 197 |
|  | |  | Cc_cds_13768_R | GAGATCTCTCCGACATTC |  |
| 35 | | *C.cajan_46779* | Cc_cds_46779_F | GTGCACCTAATGTTACCC | 191 |
|  | |  | Cc_cds_46779_R | GAGATTCTCCACGCTAGT |  |
| 36 | | *C.cajan_33873* | Cc_cds_33873_F | GTGAGAAGGATGTGATGC | 181 |
|  | |  | Cc_cds_33873_R | CTACCCATGACCAAAGAG |  |
| 37 | | *C.cajan_20342* | Cc_cds_20342_F | AGTGGGCAAGACTAGAAG | 174 |
|  | |  | Cc_cds_20342_R | CTCTCAGGTAGTCCTCCA |  |
| 38 | | *C.cajan_37860* | Cc_cds_37860_F | AGTCATGAAGAGGTACGG | 169 |
|  | |  | Cc_cds_37860_R | CCCCACAGTAAGGTAATC |  |
| 39 | | *C.cajan_30211* | Cc_cds_30211_F | GATAGTTGGAGCTAGACCAG | 153 |
|  | |  | Cc_cds_30211_R | GCTGAAGAAGACCTTGAC |  |
| 40 | | *C.cajan_17708* | Cc_cds_17708_F | CACAGTATCGCTTGACAG | 176 |
|  | |  | Cc_cds_17708_R | GTGAAGAGCCTTCTCAAC |  |
| 41 | | *C.cajan_10251* | Cc_cds_10251_F | GAGCCTGAAGAACGTAGT | 173 |
|  | |  | Cc_cds_10251_R | GGCTATACTTCTCGATGG |  |
| 42 | | *C.cajan_02901* | Cc_cds_02901_F | CCACTAGAGAAGGTACGG | 183 |
|  | |  | Cc_cds_02901_R | ACAGTGACACCCTCTGTT |  |
| 43 | | *C.cajan_20512* | Cc_cds_20512_F | CTCCTCCACGTCGATAAC | 153 |
|  | |  | Cc_cds_20512_R | GAGCTTCATCTCCTCAAG |  |
| 44 | | *C.cajan_01322* | Cc_cds_01322_F | AGGTCTCTGTCCTGGTACT | 191 |
|  | |  | Cc_cds_01322_R | GTTCTTCTGCCACCTAGT |  |
| 45 | | *C.cajan_30432* | Cc_cds_30432_F | CAAGTGTAGCAGTGTTGG | 185 |
|  | |  | Cc_cds_30432_R | TAGCTACATGGGTGAGTG |  |
| 46 | | *C.cajan_28151* | Cc_cds_28151_F | TCCTCCTCTATGTCAAGC | 156 |
|  | |  | Cc_cds_28151_R | GGCTGGCACAATAATCAC |  |
| 47 | | *C.cajan_01816* | Cc_cds_01816_F | CGCTATGGTGACATACTC | 165 |
|  | |  | Cc_cds_01816_R | GTTCTCTTCCGTGACAAC |  |
| 48 | | *C.cajan_30849* | Cc_cds_30849_F | ATCGACCTCTCCATCAAC | 187 |
|  | |  | Cc_cds_30849_R | CCTTCACGATGTGGATCT |  |
| 49 | | *C.cajan_02015* | Cc_cds_02015_F | CATGTCTACTACCACTCCAC | 162 |
|  | |  | Cc_cds_02015_R | CTTGGTTCCTTCTTGGTC |  |
| 50 | | *C.cajan_39721* | Cc_cds_39721_F | TAGGAATCCCTACCTCTC | 174 |
|  | |  | Cc_cds_39721_R | CCACCTTCAGATAGATGC |  |
| 51 | | *C.cajan_08737* | Cc_cds_08737_F | AGTCCAGCAAGGAGATAG | 196 |
|  | |  | Cc_cds_08737_R | GTTGTGGACCTCTTCTTC |  |
