# Supplementary material for: Identification and Validation of Selected Universal Stress Protein Domain Containing Drought-Responsive Genes in Pigeonpea (Cajanus cajan L.)
Source: Front Plant Sci. 2016 Jan 6;6:1065. doi: 10.3389/fpls.2015.01065 (PMC4701917; doi:10.3389/fpls.2015.01065)
Supplement: Supplementary Table 2 — List of housekeeping genes used for qRT-PCR analysis. [file Table2.DOCX]

**Supplementary Table 2.** List of housekeeping genes used for qRT-PCR analysis

| **S. No.** | | **Gene name** | | **Primer_id** | | **Primer sequence** | **Expected size (bp)** | |  |
| --- | --- | --- | --- | --- | --- | --- | --- | --- | --- |
| 1 | *Beta-tubulin* | | β-tubulin_F | | CCGTTGTGGAGCCTTACAAT | | | 117 | |
|  |  | | β-tubulin_R | | GCTTGAGGGTCCTGAAACAA | | |  | |
| 2 | *ADH* | | ADH_F | | GCTTCAAGAGCAGGTCACAAGT | | | 143 | |
|  |  | | ADH_R | | GAGACATCCTCCTTCGTGCATA | | |  | |
| 3 | *GAPDH* | | GAPDH_F | | ATGACCACCGTCCATTCCATCACT | | | 199 | |
|  |  | | GAPDH_R | | AGACATCAACAGTTGGGACACGGA | | |  | |
| 4 | *ACTIN* | | Actin 1_F | | GGCATACATTGCCCTTGACT | | | 97 | |
|  |  | | Actin 1_R | | GAACCTCGGGACATCTGAAA | | |  | |
