# Supplementary material for: Identification and Validation of Selected Universal Stress Protein Domain Containing Drought-Responsive Genes in Pigeonpea (Cajanus cajan L.)
Source: Front Plant Sci. 2016 Jan 6;6:1065. doi: 10.3389/fpls.2015.01065 (PMC4701917; doi:10.3389/fpls.2015.01065)
Supplement: Supplementary Table 3 — HMM Search output for USP domain in pigeonpea genes set. [file Table3.DOCX]

**Supplementary Table 3**: HMM Search output for USP domain in pigeonpea gene set

|  | **Full sequence** | | |  | **Best 1 domain** | | |  |
| --- | --- | --- | --- | --- | --- | --- | --- | --- |
| **S. No.** | **E-value** | **score** | **bias** |  | **E-value** | **score** | **bias** | **Genes** |
| 1 | 9.10E-032 | 111.8 | 0.1 |  | 1.00E-031 | 111.6 | 0 | *C.cajan_29409* |
| 2 | 1.10E-031 | 111.5 | 0 |  | 1.60E-031 | 111 | 0 | *C.cajan_33538* |
| 3 | 5.40E-031 | 109.3 | 0 |  | 2.20E-030 | 107.3 | 0 | *C.cajan_10251* |
| 4 | 6.40E-031 | 109.1 | 0 |  | 8.00E-031 | 108.7 | 0 | *C.cajan_28416* |
| 5 | 5.90E-029 | 102.7 | 0 |  | 7.10E-029 | 102.4 | 0 | *C.cajan_33873* |
| 6 | 9.10E-029 | 102.1 | 0.9 |  | 1.20E-028 | 101.7 | 0.6 | *C.cajan_13768* |
| 7 | 1.70E-028 | 101.2 | 0 |  | 3.20E-028 | 100.3 | 0 | *C.cajan_02504* |
| 8 | 5.60E-028 | 99.5 | 1.2 |  | 6.70E-028 | 99.3 | 0.8 | *C.cajan_25053* |
| 9 | 4.80E-027 | 96.5 | 0 |  | 5.90E-027 | 96.2 | 0 | *C.cajan_33874* |
| 10 | 3.30E-026 | 93.8 | 2.5 |  | 4.30E-026 | 93.4 | 1.8 | *C.cajan_06680* |
| 11 | 6.80E-026 | 92.8 | 0 |  | 2.50E-020 | 74.7 | 0 | *C.cajan_29830* |
| 12 | 6.00E-025 | 89.7 | 0.3 |  | 9.80E-025 | 89 | 0.2 | *C.cajan_08737* |
| 13 | 3.00E-024 | 87.4 | 0.4 |  | 3.90E-024 | 87 | 0.3 | *C.cajan_07683* |
| 14 | 1.40E-023 | 85.2 | 0 |  | 2.10E-023 | 84.7 | 0 | *C.cajan_37860* |
| 15 | 2.30E-023 | 84.5 | 0 |  | 2.90E-023 | 84.3 | 0 | *C.cajan_37861* |
| 16 | 1.90E-022 | 81.6 | 0.2 |  | 2.50E-022 | 81.2 | 0.2 | *C.cajan_23080* |
| 17 | 7.00E-022 | 79.8 | 0.3 |  | 1.30E-020 | 75.7 | 0.2 | *C.cajan_06463* |
| 18 | 7.50E-022 | 79.7 | 0.2 |  | 1.00E-021 | 79.2 | 0.1 | *C.cajan_02901* |
| 19 | 2.90E-016 | 61.6 | 0 |  | 3.50E-016 | 61.3 | 0 | *C.cajan_02015* |
| 20 | 7.00E-016 | 60.3 | 0.2 |  | 2.00E-015 | 58.8 | 0.1 | *C.cajan_30849* |
| 21 | 1.40E-015 | 59.3 | 0 |  | 2.90E-015 | 58.3 | 0 | *C.cajan_28118* |
| 22 | 3.30E-014 | 54.9 | 0 |  | 4.20E-014 | 54.5 | 0 | *C.cajan_01816* |
| 23 | 2.00E-012 | 49.1 | 1.5 |  | 5.20E-006 | 28.3 | 0 | *C.cajan_28151* |
| 24 | 5.10E-011 | 44.6 | 1 |  | 5.30E-010 | 41.3 | 0 | *C.cajan_40115* |
| 25 | 9.90E-011 | 43.6 | 0 |  | 3.80E-010 | 41.7 | 0 | *C.cajan_30432* |
| 26 | 1.90E-010 | 42.7 | 0.1 |  | 6.10E-010 | 41.1 | 0.1 | *C.cajan_20512* |
| 27 | 8.10E-010 | 40.7 | 7.4 |  | 2.10E-009 | 39.3 | 0 | *C.cajan_26230* |
| 28 | 1.80E-009 | 39.5 | 0.2 |  | 3.30E-009 | 38.7 | 0.1 | *C.cajan_31052* |
| 29 | 2.70E-009 | 39 | 0.1 |  | 7.50E-009 | 37.5 | 0.1 | *C.cajan_01322* |
| 30 | 1.30E-007 | 33.5 | 1 |  | 2.80E-007 | 32.5 | 0 | *C.cajan_03752* |
| 31 | 3.20E-007 | 32.2 | 0.2 |  | 5.90E-007 | 31.4 | 0.1 | *C.cajan_03623* |
| 32 | 3.00E-006 | 29.1 | 0 |  | 5.70E-006 | 28.2 | 0 | *C.cajan_24612* |
| 33 | 5.70E-006 | 28.2 | 0 |  | 1.00E-005 | 27.4 | 0 | *C.cajan_09181* |
| 34 | 6.50E-006 | 28 | 0.1 |  | 6.40E-005 | 24.8 | 0 | *C.cajan_16873* |
| 35 | 1.50E-005 | 26.9 | 0 |  | 1.50E-005 | 26.9 | 0 | *C.cajan_30211* |
| 36 | 1.80E-005 | 26.6 | 0 |  | 0.12 | 14.2 | 0 | *C.cajan_37347* |
| 37 | 2.20E-005 | 26.3 | 0.2 |  | 7.20E-005 | 24.6 | 0.1 | *C.cajan_20342* |
| 38 | 2.40E-005 | 26.2 | 0 |  | 2.40E-005 | 26.2 | 0 | *C.cajan_39705* |
| 39 | 2.90E-005 | 25.9 | 2.5 |  | 6.20E-005 | 24.8 | 1.7 | *C.cajan_23213* |
| 40 | 7.30E-005 | 24.6 | 1.4 |  | 0.56 | 12 | 0.1 | *C.cajan_09736* |
| 41 | 9.40E-005 | 24.3 | 0.5 |  | 0.0003 | 22.6 | 0 | *C.cajan_07270* |
| 42 | 0.00018 | 23.3 | 0 |  | 0.67 | 11.8 | 0 | *C.cajan_10348* |
| 43 | 0.00021 | 23.1 | 1.2 |  | 0.00045 | 22 | 0.8 | *C.cajan_00022* |
| 44 | 0.00032 | 22.5 | 0 |  | 0.00032 | 22.5 | 0 | *C.cajan_17708* |
| 45 | 0.00034 | 22.4 | 0 |  | 2.3 | 10 | 0 | *C.cajan_23073* |
| 46 | 0.0011 | 20.8 | 0 |  | 0.0026 | 19.6 | 0 | *C.cajan_16874* |
| 47 | 0.0012 | 20.7 | 0 |  | 2.3 | 10 | 0 | *C.cajan_46779* |
| 48 | 0.0015 | 20.4 | 0.2 |  | 0.0024 | 19.7 | 0.1 | *C.cajan_39721* |
| 49 | 0.0033 | 19.3 | 0 |  | 4.9 | 9 | 0 | *C.cajan_19839* |
| 50 | 0.0048 | 18.7 | 0 |  | 0.0048 | 18.7 | 0 | *C.cajan_20732* |
| 51 | 0.0052 | 18.6 | 0 |  | 0.011 | 17.6 | 0 | *C.cajan_08951* |
|  | ------ **inclusion threshold** ------ | | | | | | | |
| 52 | 0.013 | 17.3 | 0 |  | 0.2 | 13.4 | 0 | *C.cajan_19503* |
| 53 | 0.017 | 16.9 | 0 |  | 0.19 | 13.6 | 0 | *C.cajan_28670* |
| 54 | 0.02 | 16.7 | 0.1 |  | 8.9 | 8.1 | 0 | *C.cajan_20989* |
| 55 | 0.022 | 16.6 | 0 |  | 8.1 | 8.3 | 0 | *C.cajan_03956* |
| 56 | 0.03 | 16.1 | 0.9 |  | 6.5 | 8.6 | 0 | *C.cajan_22262* |
| 57 | 0.031 | 16.1 | 0 |  | 12 | 7.7 | 0 | *C.cajan_35964* |
| 58 | 0.065 | 15 | 0 |  | 0.087 | 14.6 | 0 | *C.cajan_12327* |
| 59 | 0.067 | 15 | 5.5 |  | 0.082 | 14.7 | 0 | *C.cajan_18267* |
| 60 | 0.099 | 14.5 | 0 |  | 0.35 | 12.7 | 0 | *C.cajan_21309* |
| 61 | 0.16 | 13.8 | 0 |  | 8.8 | 8.1 | 0 | *C.cajan_04123* |
| 62 | 0.19 | 13.5 | 0 |  | 3.7 | 9.4 | 0 | *C.cajan_05404* |
| 63 | 0.2 | 13.4 | 0 |  | 0.2 | 13.4 | 0 | *C.cajan_12261* |
| 64 | 0.22 | 13.3 | 0.8 |  | 50 | 5.7 | 0 | *C.cajan_20927* |
| 65 | 0.25 | 13.1 | 0 |  | 0.25 | 13.1 | 0 | *C.cajan_21177* |
| 66 | 0.27 | 13.1 | 0 |  | 0.38 | 12.6 | 0 | *C.cajan_01983* |
| 67 | 0.29 | 13 | 0 |  | 2.8 | 9.8 | 0 | *C.cajan_42124* |
| 68 | 0.4 | 12.5 | 0 |  | 31 | 6.4 | 0 | *C.cajan_05406* |
| 69 | 1 | 11.1 | 0 |  | 1 | 11.1 | 0 | *C.cajan_13538* |
| 70 | 1.5 | 10.7 | 2.3 |  | 21 | 6.9 | 0 | *C.cajan_12151* |
| 71 | 4.7 | 9 | 7.5 |  | 10 | 7.9 | 5.2 | *C.cajan_10092* |
