# Supplementary material for: Identification and Validation of Selected Universal Stress Protein Domain Containing Drought-Responsive Genes in Pigeonpea (Cajanus cajan L.)
Source: Front Plant Sci. 2016 Jan 6;6:1065. doi: 10.3389/fpls.2015.01065 (PMC4701917; doi:10.3389/fpls.2015.01065)
Supplement: Supplementary Table 4 — Protein names, gene ontology terms (GO_term) and ontology identities (GO_ID) of 51 drought responsive genes. [file Table4.DOCX]

**Supplementary Table 4**. Protein names, gene ontology terms (GO_term) and ontology identities (GO_ID) of 51 drought responsive genes

| **Gene_id** | **Protein_name** | **GO_term** | **GO_id** |
| --- | --- | --- | --- |
| *C.cajan_37347* | Cation/H(+) antiporter 18 (Protein CATION/H+ EXCHANGER 18) (AtCHX18) | integral component of membrane; late endosome; potassium ion transport; regulation of pH; solute: hydrogen antiporter activity | GO:0016021; GO:0005770; GO:0006813; GO:0006885; GO:0015299 |
| *C.cajan_23073* | Cation/H(+) antiporter 15 (Protein CATION/H+ EXCHANGER 15) (AtCHX15) | integral component of membrane; potassium ion transport; regulation of pH; solute: hydrogen antiporter activity | GO:0016021; GO:0006813; GO:0006885; GO:0015299 |
| *C.cajan_03623* | Probable receptor-like serine/threonine-protein kinase At5g57670 (EC 2.7.11.1) | ATP binding; protein serine/threonine kinase activity | GO:0005524; GO:0004674 |
| *C.cajan_19839* | Cation/H(+) antiporter 25 (Protein CATION/H+ EXCHANGER 25) (AtCHX25) | integral component of membrane; potassium ion transport; solute: hydrogen antiporter activity | GO:0016021; GO:0006813; GO:0015299 |
| *C.cajan_03752* | U-box domain-containing protein 34 (Plant U-box protein 34) [Includes: E3 ubiquitin ligase (EC 6.3.2.-); Serine/threonine-protein kinase (EC 2.7.11.-)] | ATP binding; protein serine/threonine kinase activity; ubiquitin ligase complex; ubiquitin-protein ligase activity | GO:0005524; GO:0004674; GO:0000151; GO:0004842 |
| *C.cajan_10348* | Cation/H(+) antiporter 28 (Protein CATION/H+ EXCHANGER 28) (AtCHX28) | integral component of membrane; membrane; potassium ion transport; solute:hydrogen antiporter activity | GO:0016021; GO:0016020; GO:0006813; GO:0015299 |
| *C.cajan_20732* | U-box domain-containing protein 33 (Plant U-box protein 33) [Includes: E3 ubiquitin ligase (EC 6.3.2.-); Serine/threonine-protein kinase (EC 2.7.11.-)] | ATP binding; protein serine/threonine kinase activity; response to stress; ubiquitin ligase complex; ubiquitin-protein ligase activity | GO:0005524; GO:0004674; GO:0006950; GO:0000151; GO:0004842 |
| *C.cajan_09736* | Cation/H(+) antiporter 20 (Protein CATION/H+ EXCHANGER 20) (AtCHX20) | cellular potassium ion homeostasis; endomembrane system; endoplasmic reticulum; integral component of membrane; potassium ion transport; protein targeting to vacuole; regulation of pH | GO:0030007; GO:0012505; GO:0005783; GO:0016021; GO:0006813; GO:0006623; GO:0006885; GO:0015299; GO:0030104 |
| *C.cajan_28416* | Universal stress protein A-like protein | plasma membrane; response to stress | GO:0005886; GO:0006950 |
| *C.cajan_37861* | Universal stress protein A-like protein | plasma membrane; response to stress | GO:0005886; GO:0006950 |
| *C.cajan_29830* | Universal stress protein A-like protein | plasma membrane; response to stress | GO:0005886; GO:0006950 |
| *C.cajan_40115* | Probable nucleoredoxin 3 (AtNrx3) (EC 1.8.1.8) | protein-disulfide reductase activity | GO:0047134 |
| *C.cajan_31052* | Receptor-like cytosolic serine/threonine-protein kinase RBK2 (EC 2.7.11.1) (Protein ROP BINDING PROTEIN KINASES 2) | ATP binding; GTPase binding; cytoplasm; nucleus; protein serine/threonine kinase activity | GO:0005524; GO:0051020; GO:0005737; GO:0005634; GO:0004674 |
| *C.cajan_08951* | Probable receptor-like serine/threonine-protein kinase At5g57670 (EC 2.7.11.1) | ATP binding; protein serine/threonine kinase activity | GO:0005524; GO:0004674 |
| *C.cajan_07683* | Universal stress protein Slr1101 (USP Slr1101) | response to stress | GO:0006950 |
| *C.cajan_06680* | Universal stress protein MJ0531 (USP MJ0531) | response to stress | GO:0006950 |
| *C.cajan_33874* | Universal stress protein A-like protein | plasma membrane; response to stress | GO:0005886; GO:0006950 |
| *C.cajan_29409* | Universal stress protein A-like protein | plasma membrane; response to stress | GO:0005886; GO:0006950 |
| *C.cajan_24612* | U-box domain-containing protein 33 (Plant U-box protein 33) [Includes: E3 ubiquitin ligase (EC 6.3.2.-); Serine/threonine-protein kinase (EC 2.7.11.-)] | ATP binding; protein serine/threonine kinase activity; response to stress; ubiquitin ligase complex; ubiquitin-protein ligase activity | GO:0005524; GO:0004674; GO:0006950; GO:0000151; GO:0004842 |
| *C.cajan_26230* | U-box domain-containing protein 35 (Plant U-box protein 35) [Includes: E3 ubiquitin ligase (EC 6.3.2.-); Serine/threonine-protein kinase (EC 2.7.11.-)] | ATP binding; protein serine/threonine kinase activity; response to stress; ubiquitin ligase complex; ubiquitin-protein ligase activity | GO:0005524; GO:0004674; GO:0006950; GO:0000151; GO:0004842 |
| *C.cajan_02504* | Universal stress protein A-like protein | plasma membrane; response to stress | GO:0005886; GO:0006950 |
| *C.cajan_39705* | U-box domain-containing protein 35 (Plant U-box protein 35) [Includes: E3 ubiquitin ligase (EC 6.3.2.-); Serine/threonine-protein kinase (EC 2.7.11.-)] | ATP binding; protein serine/threonine kinase activity; response to stress; ubiquitin ligase complex; ubiquitin-protein ligase activity | GO:0005524; GO:0004674; GO:0006950; GO:0000151; GO:0004842 |
| *C.cajan_28118* | Universal stress protein A-like protein | plasma membrane; response to stress | GO:0005886; GO:0006950 |
| *C.cajan_23080* | Universal stress protein MJ0531 (USP MJ0531) | response to stress | GO:0006950 |
| *C.cajan_06463* | Universal stress protein A-like protein | plasma membrane; response to stress | GO:0005886; GO:0006950\ |
| *C.cajan_00022* | U-box domain-containing protein 34 (Plant U-box protein 34) [Includes: E3 ubiquitin ligase (EC 6.3.2.-); Serine/threonine-protein kinase (EC 2.7.11.-)] | ATP binding; protein serine/threonine kinase activity; ubiquitin ligase complex; ubiquitin-protein ligase activity | GO:0005524; GO:0004674; GO:0000151; GO:0004842 |
| *C.cajan_09181* | U-box domain-containing protein 36 (EC 6.3.2.-) (Plant U-box protein 36) | ubiquitin ligase complex; ubiquitin-protein ligase activity | GO:0000151; GO:0004842 |
| *C.cajan_25053* | Universal stress protein A-like protein | plasma membrane; response to stress | GO:0005886; GO:0006950 |
| *C.cajan_16874* | U-box domain-containing protein 35 (Plant U-box protein 35) [Includes: E3 ubiquitin ligase (EC 6.3.2.-); Serine/threonine-protein kinase (EC 2.7.11.-)] | ATP binding; protein serine/threonine kinase activity; response to stress; ubiquitin ligase complex; ubiquitin-protein ligase activity | GO:0005524; GO:0004674; GO:0006950; GO:0000151; GO:0004842 |
| *C.cajan_16873* | U-box domain-containing protein 33 (Plant U-box protein 33) [Includes: E3 ubiquitin ligase (EC 6.3.2.-); Serine/threonine-protein kinase (EC 2.7.11.-)] | ATP binding; protein serine/threonine kinase activity; response to stress; ubiquitin ligase complex; ubiquitin-protein ligase activity | GO:0005524; GO:0004674; GO:0006950; GO:0000151; GO:0004842 |
| *C.cajan_23213* | U-box domain-containing protein 34 (Plant U-box protein 34) [Includes: E3 ubiquitin ligase (EC 6.3.2.-); Serine/threonine-protein kinase (EC 2.7.11.-)] | ATP binding; protein serine/threonine kinase activity; ubiquitin ligase complex; ubiquitin-protein ligase activity | GO:0005524; GO:0004674; GO:0000151; GO:0004842 |
| *C.cajan_33538* | Universal stress protein A-like protein | plasma membrane; response to stress | GO:0005886; GO:0006950 |
| *C.cajan_07270* | U-box domain-containing protein 33 (Plant U-box protein 33) [Includes: E3 ubiquitin ligase (EC 6.3.2.-); Serine/threonine-protein kinase (EC 2.7.11.-)] | ATP binding; protein serine/threonine kinase activity; response to stress; ubiquitin ligase complex; ubiquitin-protein ligase activity | GO:0005524; GO:0004674; GO:0006950; GO:0000151; GO:0004842 |
| *C.cajan_13768* | Universal stress protein A-like protein | plasma membrane; response to stress | GO:0005886; GO:0006950 |
| *C.cajan_46779* | Cation/H(+) antiporter 15 (Protein CATION/H+ EXCHANGER 15) (AtCHX15) | integral component of membrane; potassium ion transport; regulation of pH; solute: hydrogen antiporter activity | GO:0016021; GO:0006813; GO:0006885; GO:0015299 |
| *C.cajan_33873* | Universal stress protein A-like protein | plasma membrane; response to stress | GO:0005886; GO:0006950 |
| *C.cajan_20342* | Receptor-like cytosolic serine/threonine-protein kinase RBK2 (EC 2.7.11.1) (Protein ROP BINDING PROTEIN KINASES 2) | ATP binding; GTPase binding; cytoplasm; nucleus; protein serine/threonine kinase activity | GO:0005524; GO:0051020; GO:0005737; GO:0005634; GO:0004674 |
| *C.cajan_37860* | Universal stress protein A-like protein | plasma membrane; response to stress | GO:0005886; GO:0006950 |
| *C.cajan_30211* | U-box domain-containing protein 52 (Plant U-box protein 52) [Includes: E3 ubiquitin ligase (EC 6.3.2.-); Serine/threonine-protein kinase (EC 2.7.11.-)] | ATP binding; protein serine/threonine kinase activity; ubiquitin ligase complex; ubiquitin-protein ligase activity | GO:0005524; GO:0004674; GO:0000151; GO:0004842 |
| *C.cajan_17708* | U-box domain-containing protein 35 (Plant U-box protein 35) [Includes: E3 ubiquitin ligase (EC 6.3.2.-); Serine/threonine-protein kinase (EC 2.7.11.-)] | ATP binding; protein serine/threonine kinase activity; response to stress; ubiquitin ligase complex; ubiquitin-protein ligase activity | GO:0005524; GO:0004674; GO:0006950; GO:0000151; GO:0004842 |
| *C.cajan_10251* | Universal stress protein A-like protein | plasma membrane; response to stress | GO:0005886; GO:0006950 |
| *C.cajan_02901* | Uncharacterized protein | response to stress | GO:0006950 |
| *C.cajan_20512* | Uncharacterized protein | response to stress | GO:0006950 |
| *C.cajan_01322* | Uncharacterized protein | response to stress | GO:0006950 |
| *C.cajan_30432* | Uncharacterized protein | response to stress | GO:0006950 |
| *C.cajan_28151* | Uncharacterized protein | response to stress; vacuole | GO:0006950; GO:0005773 |
| *C.cajan_01816* | Uncharacterized protein | response to stress | GO:0006950 |
| *C.cajan_30849* | Uncharacterized protein | response to stress | GO:0006950 |
| *C.cajan_02015* | Uncharacterized protein | response to stress | GO:0006950 |
| *C.cajan_39721* | Uncharacterized protein | chloroplast stroma | GO:0009570 |
| *C.cajan_08737* | Uncharacterized protein | response to stress | GO:0006950 |
