# Supplementary material for: Identification and Validation of Selected Universal Stress Protein Domain Containing Drought-Responsive Genes in Pigeonpea (Cajanus cajan L.)
Source: Front Plant Sci. 2016 Jan 6;6:1065. doi: 10.3389/fpls.2015.01065 (PMC4701917; doi:10.3389/fpls.2015.01065)
Supplement: Supplementary Table 5 — List of genes showing more than two-fold difference between ICPL 227 and ICPL 8755. [file Table5.DOCX]

**Supplementary Table 5.** List of genes showing more than 2 fold difference between ICPL 227 and ICPL 8755

| Gene-Id | ICPL 227^$^ | ICPL 8755^$^ | Uniprot Id | Protein name |
| --- | --- | --- | --- | --- |
| *C.cajan_26230* | 5.14 | -0.40 | Q9SW11 | U-box domain-containing protein 35 |
| *C.cajan_39705* | 5.19 | -0.16 | Q9SW11 | U-box domain-containing protein 35 |
| *C.cajan_09181* | 13.50 | 0.80 | Q8GZ84 | U-box domain-containing protein 36 |
| *C.cajan_30211* | 7.13 | 1.00 | Q9FKG6 | U-box domain-containing protein 52 |
| *C.cajan_46779* | 7.47 | 0.60 | Q9SIT5 | Cation/H(+) antiporter 15 protein |
| *C.cajan_08737* | 7.70 | 0.24 | I1JEJ0 | Uncharacterized protein |
| *C.cajan_13768* | 4.56 | 0.04 | Q8LGG8 | Universal stress protein A-like protein |
| *C.cajan_23080* | 6.19 | 3.10 | Q57951 | Universal stress protein |
| *C.cajan_33538* | 4.5 | 0.97 | Q8LGG8 | Universal stress protein A-like protein |
| *C.cajan_29830* | 11.40 | 4.78 | Q8LGG8 | Universal stress protein A-like protein |
| *C.cajan_33874* | 11.68 | 5.03 | Q8LGG8 | Universal stress protein A-like protein |

**^$^** Expression fold difference between well-watered control and drought stressed plants
